# Bewegungsmangel und mögliche gesundheitliche Auswirkungen der Covid-19-Pandemie auf Kinder und Jugendliche: Was bekannt ist – und was für einen gesunden Lebensstil der Jugend zu tun wäre

**DOI:** 10.1007/s43594-022-00074-9

**Published:** 2022-09-21

**Authors:** Christine Joisten

**Affiliations:** Institut für Bewegungs- und Neurowissenschaften, Abteilung für Bewegungs- und Gesundheitsförderung, Deutsche Sporthochschule Köln, Köln, Deutschland

Heutzutage ist der Nutzen von körperlicher Aktivität insbesondere für eine gesunde Entwicklung von Kindern und Jugendlichen gut belegt. Gefordert werden von der Weltgesundheitsorganisation mindestens 60 min pro Tag mit zumindest moderater Intensität (s. u. a. Graf et al. 2017). Für Deutschland erfolgte eine Zusammenstellung im Jahr 2015 infolge einer Recherche und Bewertung aller bis zu diesem Zeitpunkt erschienenen internationalen Empfehlungen beziehungsweise systematischen Reviews unter Berücksichtigung verschiedener Altersgruppen; integriert wurden außerdem die Ergebnisse eines Expertenkonsens aus 2012 (Graf et al. 2014) (siehe Tab. 1). Generell zeichneten sich aber in den vergangenen Jahrzehnten ein deutlicher Rückgang von Bewegung und die Zunahme vermeidbarer Sitzzeiten ab. Zusätzlich wurde in den vergangenen Jahrzehnten ein signifikanter Rückgang der körperlichen Aktivität sowie der kardiovaskulären und motorischen Fitness von Kindern beobachtet (Schlag et al. 2021).

**Table 1:** 

**Bewegung**	
*Säuglinge und Kleinkinder*	Säuglinge und Kleinkinder sollten so wenig wie möglich in ihrem natürlichen Bewegungsdrang gehindert werden und sich so viel wie möglich bewegen; auf sichere Umgebungsbedingungen ist zu achten
*Kindergartenkinder* (4 bis 6 Jahre)	Für Kindergartenkinder soll eine angeleitete und nichtangeleitete Bewegungszeit von 180 min/Tag und mehr erreicht werden
*Grundschulkinder* (6 bis 11 Jahre)	Für Kinder ab dem Grundschulalter soll eine tägliche Bewegungszeit von 90 min und mehr mit moderater^a^ bis intensiver^b^ Intensität erreicht werden
	60 min davon können durch Alltagsaktivitäten, zum Beispiel Schulweg, absolviert werden, jedoch mindestens 12.000 Schritte/Tag
*Jugendliche* (12 bis 18 Jahre)	Für Jugendliche soll eine tägliche Bewegungszeit von 90 min und mehr mit moderater bis intensiver Intensität erreicht werden
	60 min davon können durch Alltagsaktivitäten, zum Beispiel mindestens 12.000 Schritte/Tag, absolviert werden
*Spezifische Aspekte*	
Besonderheiten, aber auch Neigungen, Bedürfnisse und mögliche Barrieren der jeweiligen Zielgruppe, zum Beispiel Alter, Geschlecht, soziokulturelle Faktoren, sollen berücksichtigt werden	
Allgemein soll eine Förderung der motorischen Leistungsfähigkeit alters- und geschlechtsangepasst durchgeführt werden	
Ab dem Grundschulalter soll zur Verbesserung von Kraft und Ausdauer an zwei bis drei Tagen pro Woche eine intensive Beanspruchung der großen Muskelgruppen erfolgen, jeweils unter Berücksichtigung des individuellen Entwicklungsstandes	
„Bewegungsarme“ Kinder und Jugendliche sollten schrittweise an das Ziel herangeführt werden, zum Beispiel durch zunächst 30 min Bewegung an ein bis zwei Tagen pro Woche, anschließend werden der zeitliche Umfang, dann die Intensität gesteigert	
**Sitzende Tätigkeiten in der Freizeit/Bildschirmmedien**	
*Vermeidbare Sitzzeiten sollten auf ein Minimum reduziert werden. Neben (motorisiertem) Transport, zum Beispiel in Babyschale oder Kindersitz, oder unnötig im Haus verbrachten Zeiten, betrifft dies insbesondere die Reduktion des Bildschirmmedienkonsums auf ein Minimum:*	
Säuglinge und Kleinkinder: 0 min	
Kindergartenkinder: möglichst wenig, maximal 30 min/Tag	
Grundschulkinder: möglichst wenig, maximal 60 min/Tag	
Jugendliche: möglichst wenig, maximal 120 min/Tag	

^a^Moderate Intensität entspricht einer leichten Steigerung der Herzfrequenz beziehungsweise etwas angeregterer Atmung (siehe auch http://www.cdc.gov/physicalactivity/basics/children/)

^b^Intensive Intensität entspricht einer deutlichen Steigerung der Herzfrequenz beziehungsweise erheblich angeregterer Atmung (http://www.cdc.gov/physicalactivity/basics/children)

Seit Beginn der Covid-19-Pandemie wurde und wird dieser negative Trend durch Schulschließungen, den Wegfall von Sportunterricht, Fernunterricht verbunden mit dem Mangel an aktiven Schulwegen, durch die Schließung öffentlicher Sportstätten (Abb. 1) und das Fehlen von organisierten Sportangeboten deutlich verstärkt (Adamakis 2021). So beschrieben Rossi et al. (2021) in einer Übersichtsarbeit einen Rückgang von 45 bis 91 min pro Tag im Vergleich zu vor der Pandemie. Allerdings liegen nach wie vor kaum einheitlich erfasste und repräsentative Daten vor. Auf der Grundlage von Daten aus dem Motorik-Modul beziehungsweise der MoMo-Studie als Teil des Kinder- und Jugendgesundheitssurveys stieg in Deutschland die tägliche körperliche Aktivität während des ersten Lockdowns (März bis Juni 2020) zunächst von 108,0 auf 146,8 min pro Tag, sank dann aber während des zweiten Lockdowns (November 2020 bis Januar 2021) auf 62,2 min (Schmidt et al. 2021). Wenig überraschend ist ein deutlicher Anstieg des Medienkonsums (in der Freizeit), teils von einer auf bis zu vier Stunden pro Tag (ten Velde et al. 2021). Besonders deutlich wurden diese Entwicklungen in Familien mit einem niedrigeren sozioökonomischen Status (SES) beobachtet; sie hatten weniger Zugang zu Freiflächen, und folglich hatten die Kinder auch weniger Möglichkeiten, sich körperlich zu betätigen (Perez et al. 2021).

**Figure 1:**
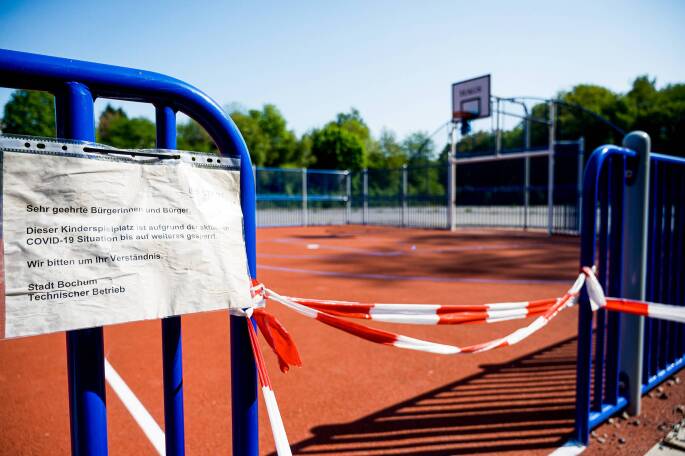

Im Allgemeinen werden weniger körperliche Aktivität und mehr Bildschirmzeit mit der Entstehung von Übergewicht beziehungsweise mit dem Auftreten kardiometabolischer Risikofaktoren, schlechter körperlicher Leistungsfähigkeit und schlechterer psychischer Gesundheit in Verbindung gebracht. Auch dieser Zusammenhang wurde beziehungsweise wird infolge der Covid-19-Pandemie verstärkt (Carson et al. 2016; Tandon et al. 2021). So zeigten beispielsweise Musa et al. (2022) in einer systematischen Analyse, dass die Bildschirmmedienzeit mit einem deutlich höheren Auftreten von Komponenten des metabolischen Syndroms vergesellschaftet war. In diesem Beitrag sollen nun die bisher bekannten allgemeinen gesundheitlichen Folgen der Covid-19-Pandemie im Kindes- und Jugendalter unter besonderer Berücksichtigung von Bewegung beleuchtet werden.

## Gesundheitliche Folgen der Covid-19-Pandemie

Seit die Weltgesundheitsorganisation im März 2020 SARS-CoV‑2 zur Pandemie erklärt hat, wurden und werden zahlreiche Untersuchungen durchgeführt, wie sich die verschiedenen Maßnahmen auf vulnerable Gruppen auswirkten. Dazu werden insbesondere auch Kinder und Jugendliche gezählt. Medial wurden und werden (wieder einmal) die Kinder als „verlorene Generation“, „Verlierer in der Pandemie“ tituliert; sie seien unglücklicher und dicker geworden. Ob dem wirklich so ist, kann aktuell (noch) gar nicht beantwortet werden, da die Datenlage zwar umfangreich ist, aber meist methodische Schwächen aufweist. So wurden überwiegend Querschnittsanalysen und/oder Befragungen durchgeführt. Das soll die mögliche Problematik nicht schmälern, aber in ein differenziertes Licht rücken.

Unstrittig ist, dass sich – je nach Pandemiephase – der gewohnte Alltag von Kindern und Jugendlichen erheblich änderte. Das Schließen von Kindergärten und Schulen, Freizeitangeboten sowie die mit dieser Pandemiesituation verbundene Ungewissheit, die im März 2020 nahezu unvermittelt heftig eintrat, haben zu einem enormen Anstieg negativer Reaktionen geführt (Camoirano 2017). In Deutschland hat die Corona-und-Psyche(COPSY)-Studie gezeigt, dass sich während der ersten Welle der Pandemie im Frühjahr 2020 71 % der Kinder und Jugendlichen gestresst fühlten – durch die Verlagerung des Schulbetriebs auf den Hausunterricht (64,4 %), den verringerten Kontakt zu Freund*innen (82,2 %) und häufigere Streitigkeiten in der Familie (27,6 %) (Ravens-Sieberer et al. 2021). Darüber hinaus verdoppelte sich die Prävalenz von Kindern und Jugendlichen mit psychischen Problemen fast, von 17,6 % (*n* = 273) auf 30,4 % (*n* = 482).

Viner et al. (2022) integrierten 36 Studien aus elf Ländern in ein systematisches Review, in dem ebenfalls die Effekte von Schulschließungen und Lockdowns während der ersten Covid-19 Welle analysiert wurden. Zusammenfassend habe infolgedessen der Zugang zu schulischen Dienstleistungen wie Lern- und Entwicklungsraum, auch zu Schulessen und Schulgesundheitsdiensten gefehlt. So böten Schulen einen Schutzraum gegenüber möglicher Vernachlässigung, Missbrauch und/oder Gewalt. Der Mangel an sozialen Kontakten und gewohnten Strukturen, geschätzten Aktivitäten, inklusive auch dem aktiven Schulweg, mag ebenfalls mit negativen Symptomen der psychischen Gesundheit (wie zum Beispiel Stress und Angst) und Gesundheitsverhalten (beispielsweise mehr Bildschirmnutzung und geringere körperliche Aktivität) in Verbindung stehen. Trotz methodischer Schwächen in den integrierten Studien zeigte sich übereinstimmend eine schlechtere psychische Gesundheit und Wohlbefinden. 18 bis 60 % der Kinder und Jugendlichen lag über den jeweiligen Schwellenwerten für das Risiko psychischer Störungen. Parallel zeigten sich ein Rückgang von 50 bis 65 % bei den Behandlungen wegen Selbstverletzungen und 40 % weniger psychiatrische Einweisungen. Laut der Autor*innen deutete dieser Kontrast zwischen dem Anstieg des Leidensdrucks und dem Rückgang der Einweisungen auf einen ungedeckten Bedarf hin, insbesondere bei bereits gefährdeten Kindern und Jugendlichen. Noch beunruhigender waren aus Sicht der Autor*innen die Belege für eine Verringerung der Fähigkeit der Gesundheits- und Sozialfürsorgesysteme zum Schutz der Kinder in vielen Ländern.

Hinzu kamen Ängste vor einer eigenen Erkrankung oder einer Transmission auf Freund*innen und (ältere) Familienmitglieder sowie möglichen Verluste und eine entsprechend eher negativ geprägte Kommunikation in Familien (Uy et al. 2022). Diese Belastungen sowie der vorherrschende Stressfaktor wurden und werden als zentrale Einflussfaktoren auf den Lebensstil und gesundheitliche Parameter gesehen. Dieser wirkte sich nicht nur auf das Ess- und Bewegungsverhalten beziehungsweise die psychosozialen Belastungen aus. In einer Übersichtsarbeit von Mayra et al. (2022) wurden in nahezu allen integrierten Studien auch Veränderungen der Schlafgewohnheiten beobachtet, wobei über spätere Schlaf- und Wachzeiten sowie Schwierigkeiten bei der Einleitung und Aufrechterhaltung des Schlafs in verschiedenen Bevölkerungsgruppen (zum Beispiel Kinder mit Übergewicht oder Adipositas sowie Jugendliche mit oder ohne ADHS) berichtet wurde.

In einer eigenen Untersuchung gaben Eltern die höchsten psychischen Belastungswerte bei Kindern im Grundschulalter an, meist aufgrund von dem Gefühl der Einsamkeit und verbunden mit der Quarantäne von mindestens einem Familienmitglied. Eine höhere psychische Belastung der Kinder und Jugendlichen korrelierte mit einem höheren Stressempfinden beziehungsweise schlechteren Bewältigungsstrategien der Eltern (Nöthig et al. 2022). Diese bewerteten die mit der Pandemie verbundenen Schul- und Kindergartenschließungen, fehlenden soziale Kontakte und Freizeitaktivitäten überwiegend negativ, außer in der Gruppe der 14- bis unter 16-Jährigen. Möglicherweise haben Jugendliche bereits unabhängige Bewältigungsstrategien entwickelt, wie zum Beispiel den Kontakt mit anderen über Online-Plattformen, einschließlich gemeinsamem Videospielen und der Nutzung sozialer Medien.

Auch in der COSMO-Studie wiesen Eltern mit schulpflichtigen Kindern eine signifikant höhere psychische Belastung im Vergleich zur allgemeinen Studienpopulation auf, insbesondere während der der ersten Pandemiewelle von März 2020 bis Mai 2020 (Germany COVID-19 Snapshot MOnitoring (COSMO Germany). „Questionnaire wave 4, 24 March 2020–25 March 2020“. Available online: https://dfncloud.uni-erfurt.de/s/Cmzfw8fPRAgzEpA#pdfviewer (zugegriffen: 24. Juli 2021)). Als eine mögliche Folge könnten Störungen in den familiären Beziehungen ihrerseits wiederum die psychische Gesundheit der Kinder negativ beeinflusst haben. Eine Untersuchung über besondere Stressfaktoren bei Eltern von Kindern im Alter von 0 bis 12 Jahren während der Covid-19-Pandemie im Vereinigten Königreich zeigte Schwierigkeiten bei der Bewältigung der verschiedenen Aufgaben im Haushalt und bei der Anpassung der bisherigen Strukturen und Routinen des häuslichen Lebens an die neue Situation (Dawes et al. 2021). Darüber hinaus entfielen häufig die üblichen Unterstützungsnetze und persönlichen Beziehungen. Mütter und Alleinerziehende erwiesen sich als besonders gefährdete Gruppe. Infolgedessen forderten die Autor*innen die politischen Entscheidungsträger*innen und Arbeitgeber*innen auf, geeignete adaptive Bewältigungsstrategien zu fördern, um Eltern und damit auch ihre Kinder während der Pandemie zu unterstützen, zum Beispiel durch Beratungsangebote bei psychischen Belastungen, Strategien zum Umgang mit Einsamkeit und Hilfe bei der Kinderbetreuung. Darüber hinaus sollten Eltern hinsichtlich einer angemessenen Kommunikation, psychischen Gesundheit, Hygiene, adaptiven Bewältigungsstilen als positive Vorbilder und das Üben adaptiver Bewältigung unterstützt werden.

## Was Eltern über die Gesundheit ihrer Kinder sagen

**Figure 2:**
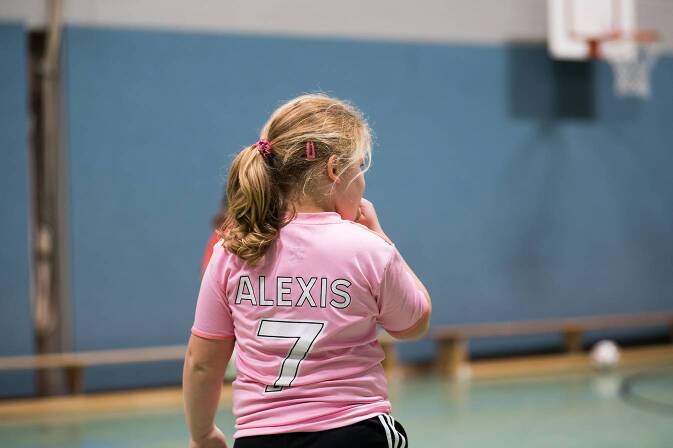

Im Frühjahr 2022 startete die Deutsche Adipositas Gesellschaft mit der Arbeitsgemeinschaft Adipositas im Kindes- und Jugendalter und dem Meinungsforschungsinstitut Forsa im März und April 2022 eine Panelumfrage mit 1004 Eltern mit Kindern im Alter von 3 bis 17 Jahren (Weihrauch-Blüher et al. in Vorbereitung). 43 % der Kinder und Jugendlichen wiesen eine „mittlere“ oder „starke“ psychische Belastung auf. 16 % der Kinder und Jugendlichen nahmen zu, in der Altersgruppe der 10- bis 12-Jährigen waren es 32 %. Besonders betroffen waren auch hier Kinder und Jugendliche aus einkommensschwachen Familien (23 zu 12 %). 44 % der Kinder und Jugendlichen bewegten sich insgesamt weniger als vor der Pandemie (Abb. 2). Auch in diesem Punkt war die Altersgruppe 10 bis 12 Jahre mit 57 % häufiger betroffen. Die körperliche Fitness nahm dementsprechend bei 33 % aller Kinder und Jugendlichen beziehungsweise 48 % bei den 10- bis 12-Jährigen ab. 70 % der Kinder und Jugendlichen steigerten ihre Mediennutzung; 27 % konsumierten häufiger Süßwaren. Positiv aber wurde vermerkt, dass 34 % der Familien häufiger zusammen aßen. Somit gab es durchaus auch günstige Effekte. So beschrieben Ilesanmi et al. (2021) auf Basis von 17 Studien einen Rückgang des Konsums von Fast Food sowie einen vermehrten Konsum an Obst und Gemüse und mehr Zeit mit der Familie zu Hause. Teilweise wurde mehr Sport getrieben und das sitzende Verhalten sogar reduziert. Dies wurde von den Autor*innen unter anderem auf die höhere Verfügbarkeit an Zeit durch das Home-Schooling zurückgeführt, die wiederum aktiv genutzt wurde. Auch der Online-Unterricht führte bei Jugendlichen im Alter von 10 bis 15 Jahren im Vergleich zu Jugendlichen im Alter von 16 bis 19 Jahren zu besseren schulischen Leistungen. Außerdem wurde ein Rückgang akuter Alkoholintoxikationen beschrieben (Pigeaud et al. 2021). Zurückgeführt wurde diese Beobachtung unter anderem auf die Schließung von Bars/Restaurants, Sportvereinen und Schulen sowie durch die verstärkte elterliche Aufsicht aufgrund von (obligatorischem) Home-Office.

## Diskussion und Fazit

Zusammenfassend zeigt sich ein sehr heterogenes Bild bezüglich der bisher bekannten gesundheitlichen Folgen der Covid-19-Pandemie. Der Schwerpunkt der Forschungen sind psychosoziale, emotionale Erscheinungen sowie Änderungen des Lebensstils (Xiang et al. 2020; Almeida et al. 2021). Welche Konsequenzen sich tatsächlich langfristig daraus ergeben, bleibt abzuwarten. Dabei ist sicherlich eine Förderung von Bewegung und Reduktion vermeidbarerer Sitz- und insbesondere Medienzeiten auch weiterhin aus den eingangs genannten Gründen sinnvoll. Dabei handelt es sich allerdings um keine unbekannte Forderung; das „Problem“ wurde durch die Pandemie „nur“ verschärft. Analog sind die sozialen Unterschiede pandemiebedingt nicht nur offensichtlicher, sondern (noch) größer geworden (Koletzko et al. 2021). Wessely et al. (2022) zeigten in drei Kohorten aus der Zeit vor (2016) und während der Pandemie (2020, 2021) eine signifikante Abnahme der motorischen Leistungsfähigkeit, vor allem bei Kindern mit einer hohen sozialen Belastung, sowie einen Anstieg von Übergewicht und Adipositas.

Neben finanziellen Unterstützungen in (besonders) bedürftigen Familien ist die Förderung eines gesunden Lebensstils durch eine entsprechende Gestaltung von Lebenswelten und Bewegungsräumen mit Parkanlagen, Grünflächen, Spielplätzen et cetera im Sinne einer gesteigerten Moveability sinnvoll (Padial-Ruz et al. 2021). Auch wenn verhältnispräventive Interventionen am ehesten erfolgversprechend sind, werden Maßnahmen im Bereich der öffentlichen Gesundheit oft unzureichend finanziert. Das betrifft in der Regel sowohl den Zeitrahmen der jeweiligen Projekte als auch eine begleitende Evaluierung (Magnusson et al. 2014). Nicht selten führt das zu einem Scheitern oder einem Mangel an Nachhaltigkeit erfolgversprechender Maßnahmen. Daher sollten auch diese Punkte verstärkt in den Blick genommen werden, nicht nur um den pandemiebeförderten gesundheitlichen Risiken entgegenzuwirken, sondern bestenfalls auch die schon lange geforderte Trendwende zu erzielen. Eine Möglichkeit der Herangehensweise bietet der Ansatz der gemeinschaftsbasierten partizipativen Forschung (Community based participatory research/CBPR; CBPR nach Israel et al. 1998; Minkler und Wallerstein 2003). Dieser Ansatz stellt weniger eine Forschungsmethode als eine partizipative Herangehensweise von Vertreter*innen aus Wissenschaft, Praxis, Bevölkerung und Kommune dar, um Ursachen von gesundheitlichen, hier pandemiebedingten/-verschärften Risiken zu erforschen und gemeinsam Handlungsstrategien zu entwickeln (mod. nach Israel et al. 2003). Im Fokus solcher Bestrebungen stehen häufig sozial benachteiligte Quartiere und Kommunen, die von dieser Zusammenarbeit im Sinne der Befähigung zu individueller und kollektiver Selbstbestimmung (Empowerment) und Kompetenzentwicklung (Capacity Building) profitieren. Damit soll letztlich die Basis für eine langfristige Zusammenarbeit und die Verankerung der jeweiligen (gesundheitlichen) Themen als Querschnittsaufgabe aller beteiligten Partner*innen geschaffen werden.

Es bleibt daher zu hoffen, dass die Besonderheiten der Covid-19-Pandemie nicht nur ein Strohfeuer des Aktionismus entfacht haben, das mit der „politischen“ Gießkanne gelöscht wird, sondern fest verankerte Strukturen zur Förderung gesundheitlicher Chancengleichheit für besonders belastete Gruppen, unter anderem Kinder und Jugendliche, im Sinne von Health-in-all-Policies entstehen.
